# Gut microbiota dysbiosis in male patients with chronic traumatic complete spinal cord injury

**DOI:** 10.1186/s12967-018-1735-9

**Published:** 2018-12-13

**Authors:** Chao Zhang, Wenhao Zhang, Jie Zhang, Yingli Jing, Mingliang Yang, Liangjie Du, Feng Gao, Huiming Gong, Liang Chen, Jun Li, Hongwei Liu, Chuan Qin, Yanmei Jia, Jiali Qiao, Bo Wei, Yan Yu, Hongjun Zhou, Zhizhong Liu, Degang Yang, Jianjun Li

**Affiliations:** 1 School of Rehabilitation Medicine, Capital Medical University, No. 10 Jiaomen North Road, Fengtai District, Beijing, 100068 China; 2 Department of Spinal and Neural Function Reconstruction, China Rehabilitation Research Center, Beijing, 100068 China; 3 Center of Neural Injury and Repair, Beijing Institute for Brain Disorders, Beijing, 100068 China; 4 China Rehabilitation Science Institute, Beijing, 100068 China; 5 Beijing Key Laboratory of Neural Injury and Rehabilitation, Beijing, 100068 China; 6 Institute of Rehabilitation Medicine, China Rehabilitation Research Center, Beijing, 100068 China; 7 Department of Spinal Cord Injury Rehabilitation, China Rehabilitation Research Center, Beijing, 100068 China; 8 Laboratory Medicine, China Rehabilitation Research Center, Beijing, 100068 China

**Keywords:** Gut microbiota dysbiosis, Chronic traumatic complete SCI, Neurogenic bowel management, NBD symptoms, Serum biomarkers

## Abstract

**Background:**

Neurogenic bowel dysfunction (NBD) is a major physical and psychological problem in patients with spinal cord injury (SCI), and gut dysbiosis is commonly occurs in SCI. Here, we document neurogenic bowel management of male patients with chronic traumatic complete SCI in our centre and perform comparative analysis of the gut microbiota between our patients and healthy males.

**Methods:**

A total of 43 male patients with chronic traumatic complete SCI (20 with quadriplegia and 23 with paraplegia) and 23 healthy male adults were enrolled. Clinical data and fresh stool specimens were collected from all participants. Face-to-face interviews were conducted to survey the neurogenic bowel management of 43 patients with SCI. Gut microbiomes were analysed by sequencing of the V3–V4 region of the 16S rRNA gene.

**Results:**

NBD was common in adult male patients with chronic traumatic complete SCI. Patients with quadriplegia exhibited a longer time to defecate than did those with paraplegia and had higher NBD scores and heavier neurogenic bowel symptoms. The diversity of the gut microbiota in the SCI group was reduced, and the structural composition was different from that of the healthy adult male group. The abundance of Veillonellaceae and Prevotellaceae increased, while Bacteroidaceae and Bacteroides decreased in the SCI group. The abundance of Bacteroidaceae and Bacteroides in the quadriplegia group and Acidaminococcaceae, Blautia, Porphyromonadaceae, and Lachnoclostridium in the paraplegia group were significantly higher than those in the healthy male group. Serum biomarkers (GLU, HDL, CR, and CRP), NBD defecation time and COURSE had significant correlations with microbial community structure. Microbial community structure was significantly associated with serum biomarkers (GLU, HDL, CR, and CRP), NBD defecation time, and COURSE.

**Conclusions:**

This study presents a comprehensive landscape of the gut microbiota in adult male patients with chronic traumatic complete SCI and documents their neurogenic bowel management. Gut microbiota dysbiosis in SCI patients was correlated with serum biomarkers and NBD symptoms.

**Electronic supplementary material:**

The online version of this article (10.1186/s12967-018-1735-9) contains supplementary material, which is available to authorized users.

## Background

After complete spinal cord injury (SCI), the loss of descending control over sympathetic preganglionic neurons causes the autonomic reflex circuitry to become dysfunctional, creating pathology including autonomic dysreflexia and SCI–immune depression syndrome [1–5]. This dysfunction causes an autonomic imbalance in the gastrointestinal tract, which leads to deficits in colonic motility, mucosal secretions, and vascular tone [6, 7]. The early survival rate of these patients has significantly improved, but the quality of life of these patients remains unsatisfactory. Among the issues facing patients with SCI, neurogenic bowel dysfunction (NBD) is a major physical and psychological problem that can seriously affect the quality of life. The two main manifestations of NBD are constipation and faecal incontinence; the reported prevalence of constipation in these patients is 40–58%, and faecal incontinence ranges from 2 to 61% [8–11]. Because of these problems, compared with matched controls, patients with chronic SCI tend to spend more time in the toilet while evacuating their bowels; use suppositories, laxatives and supplemental dietary fibre more frequently to improve bowel evacuation; and require manual removal of faeces much more frequently [12–15]. One of the aims of our present study was to document neurogenic bowel management of male patients with chronic traumatic complete SCI in our centre.

The human intestinal tract is colonised by thousands of different genera of bacterial species whose number and genetic content exceed that of the host by a factor of ten- and 150-fold, respectively [16]. These bacteria are critical for normal digestion; nutrient absorption; and the development, metabolism, and function of cells throughout the body [17, 18]. Based on recent studies, an imbalance of the normal gut microbiota (dysbiosis) is associated with inflammatory bowel diseases [19], irritable bowel syndrome and other diseases [20, 21].

Common causes of gut dysbiosis include antibiotic use, prolonged stress, and gastrointestinal dysfunction [17, 22, 23]. Most patients with acute complete SCI have an altered intestinal transit time and loss of intestinal mucosal function, resulting in the displacement of intestinal flora in the intestines. The use of antibiotics also affects healthy intestinal micro-ecological systems [24–27]. Moreover, gut dysbiosis is likely to occur in SCI.

There are few clinical studies on intestinal microecology after SCI. Using stool samples from traumatic SCI mice, Kigerl et al. showed that traumatic SCI can cause intestinal disorders and that dysbiosis can impair functional recovery [28]. Bilgi et al. reported a clinical study on 30 patients with SCI and showed that the number of butyrate-producing communities in patients with SCI was significantly lower than that in normal controls [27]. However, more studies are needed to determine whether intestinal dysbiosis after SCI results in changes in a range of clinically relevant variables [29].

The aims of this article were to document neurogenic bowel management of male patients with chronic traumatic complete SCI in our centre, perform comparative analysis of intestinal gut microbiota in male patients with chronic traumatic complete SCI and healthy males and explore the association between intestinal microbiota with serum biomarkers and neurogenic bowel symptoms.

## Materials and methods

### Ethics statement

Approval of the hospital ethics committee was obtained before commencing the study.

### Patients and controls

A total of 43 male patients with chronic traumatic complete SCI (20 with quadriplegia and 23 with paraplegia) were enrolled in our centre from March 2017 to October 2017 using a face-to-face clinical questionnaire survey. Signed informed consent was obtained before the assessment, and we used the “International Spinal Cord Injury Core Data Set”, “International bowel function basic spinal cord injury data set” and “International bowel function extended spinal cord injury data set” to obtain NBD symptom dates [30–32].

Patients were included if they met the following criteria: (1) neurologically complete SCI (ASIA grade A) occurring 6 or more months prior to the study, (2) 18–60 years of age, (3) traumatic SCI, and (4) male. The diagnostic criteria for patients with chronic traumatic complete SCI included 1 and 3 of the above criteria. The exclusion criteria were as follows: (1) inability to complete a questionnaire survey; (2) a history of antibiotic or probiotic use in the first month before enrolment; and (3) diabetes, gastrointestinal system diseases, multiple sclerosis, and immune metabolic diseases.

A total of 43 patients with SCI and 23 healthy male adults were enrolled in this study. The clinical dates and fresh stool specimens of the subjects were collected. After extracting faecal genomic DNA, the V3–V4 region of 16S rDNA was amplified, and an Illumina MiSeq platform was used to analyse and compare the gut microbiota of healthy males with that of male patients with SCI.

The criteria for the healthy control group were as follows: (1) 18–60 years of age; (2) no history of antibiotics or probiotics use 1 month prior to study; and (3) no history of diabetes, gastrointestinal system diseases, multiple sclerosis, and immune metabolic diseases. All subjects were selected before sampling and signed informed consent with a full understanding of the sampling process and research options. All patients and healthy subjects were fed standard hospital food 2 weeks before stool collection to exclude the potential effects of diet on the gut microbiota.

### Microbial diversity analysis

#### Stool sampling

A total of 66 fresh specimens were collected, including 23 healthy males and 43 patients with SCI. Fresh faecal samples were collected and transferred to the laboratory. Samples were placed in a new 2-mL sterile centrifuge tube, quickly placed on ice, and transferred to a freezer maintained at − 80 °C for cryopreservation. The entire sampling process was completed in 30 min.

#### DNA extraction and PCR amplification

Microbial DNA was extracted from stool samples using an E.Z.N.A.^®^ Stool DNA Kit (Omega Bio-tek, Norcross, GA, U.S.) according to the manufacturer’s protocols. The V3–V4 region of the bacterial 16S rRNA gene was amplified by PCR (95 °C for 2 min, followed by 25 cycles at 95 °C for 30 s, 55 °C for 30 s, and 72 °C for 30 s and a final extension at 72 °C for 5 min) using primers 338F 5′-ACTCCTACGGGAGGCAGCA-3′ and 806R 5′-GGACTACHVGGGTWTCTAAT-3′. PCRs were performed in triplicate in a 20 μL mixture containing 4 μL 5× FastPfu Buffer, 2 μL 2.5 mM dNTPs, 0.8 μL of each primer (5 μM), 0.4 μL FastPfu Polymerase, and 10 ng template DNA.

#### Illumina MiSeq sequencing

Amplicons were extracted from 2% agarose gels, purified by using an AxyPrep DNA Gel Extraction Kit (Axygen Biosciences, Union City, CA, U.S.) and quantified by using QuantiFluor™-ST (Promega, U.S.). Purified amplicons were pooled in equimolar concentrations and paired-end sequenced (2 × 300 bp) on an Illumina MiSeq platform according to the standard protocols. The library construction steps are as follows: (1) connecting the Y-shaped linker; (2) using the magnetic bead to remove the self-ligated fragment of the linker; (3) enriching the library template by PCR amplification; and (4) denaturing sodium hydroxide, producing single-stranded DNA fragments. The raw reads were deposited into the NCBI Sequence Read Archive database (Accession Number: SRP158549).

#### Processing of sequencing data

Raw fastq files were quality-filtered by Trimmomatic and merged by FLASH with the following criteria: (i) the reads were truncated at any site receiving an average quality score < 20 over a 50 bp sliding window. (ii) Sequences whose overlap was longer than 10 bp were merged according to their overlap with no more than 2 mismatched bp. (iii) The sequences of each sample were separated according to barcodes (exactly matching) and primers (allowing 2 mismatching nucleotides), and reads containing ambiguous bases were removed.

Operational taxonomic units (OTUs) were clustered with 97% similarity cutoff using UPARSE (version 7.1), and chimeric sequences were identified and removed using UCHIME. The taxonomy of each 16S rRNA gene sequence was analysed by the RDP Classifier algorithm against the Silva (SSU123) 16S rRNA database using a confidence threshold of 70% Roche 454 (Roche, Switzerland). High-throughput sequencing of the PCR products was performed by Shanghai Majorbio Biological Technology Co. Ltd., Shanghai, China.

### Bioinformatic and statistical analysis

Sequencing reads were processed using QIIME (version 1.9.0) and included additional quality trimming, demultiplexing, and taxonomic assignments. Profiling of predictive urine microbiota was performed by using PiCRUSt based on the 13 August 2013 Greengenes database [33]. The KW rank sum test and pairwise Wilcoxon test were used for the identification of the different markers, and LDA was used to score each feature in LEfSe analysis. The index of alpha diversity was calculated with QIIME based on sequence similarity of 97%. Beta diversity was measured by unweighted UniFrac distance, which was also calculated by QIIME. Hierarchical clustering was performed, and a heatmap was generated using Spearman’s rank correlation coefficient as a distance measure and a customised script developed in the R statistical package. The output file was further analysed using the Statistical Analysis of Metagenomic Profiles software package (version 2.1.3) [34].

Statistical analysis was performed using the SPSS data analysis program (version 21.0) and Statistical Analysis of Metagenomic Profiles software. For continuous variables, independent t-tests, Welch’s t-tests, White’s nonparametric t-tests, and Mann–Whitney U-tests were applied. For categorical variables between groups, we used either the Pearson Chi square or Fisher’s exact test, depending on assumption validity. For taxa among subgroups, ANOVA was applied (Tukey–Kramer post hoc tests were used, and the effect size was Eta-squared) with Benjamini–Hochberg FDP false discovery rate correction [34, 35]. All tests of significance were two-sided and p < 0.05.

## Results

### Characteristics and neurogenic bowel management of male patients with chronic traumatic complete SCI

In total, 43 patients with chronic SCI fulfilling the enrolment criteria were interviewed and completed the survey form (Table 1). The causes of injury in descending order of frequency were traffic accidents (37.2%), bruised by heavy object (20.9%), and fall from height (20.9%). The mean NBD score was 10.02 ± 5.11. The mean defecation time was 35.33 ± 16.766 min. Most patients (60.5%) engaged in bowel care more than twice every week, and the remaining patients (39.5%) engaged in bowel care once daily. The main techniques used for faecal evacuation in descending order of frequency were suppository (88.4%), manual evacuation (23.3%), digital stimulation (16.3%), and spontaneous evacuation (4.7%). Supplementary interventions for faecal evacuation included abdominal massage (58.1%), digital anus–rectal stimulation (48.8%), digital evacuation (9.4%), and cathartic drug use (9.4%).

**Table 1:** Neurogenic bowel management table in male patients with chronic traumatic complete SCI

	SCI-male n (%)	SCI-cervical n (%)	SCI-thoracic and lumbar n (%)	p
Course	62.5 ± 53.98	69.4 ± 52.72	56.5 ± 54.36	0.449
NBD scores	10.02 ± 5.11	11.17 ± 5.16	8.7 ± 4.72	0.119
Defecation time	35.33 ± 16.766	41.789 ± 19.29	30 ± 13.94	0.026
Pathogenesis	Traffic accident 16 (37.2%) Bruised by heavy object 9 (20.9%) Falling down 9 (20.9%) Other causes 9 (20.9%)	Traffic accident 10 (50%) Bruised by heavy object 4 (20%) Falling down 3 (15%) Other causes 3 (15%)	Traffic accident 6 (26.1%) Bruised by heavy object 5 (21.7%) Falling down 6 (26.1%) Other causes 6 (26.1%)	
Frequency of bowel care	Once a day 17 (39.5%) Not daily but more than twice every week 26 (60.5%)	Once daily 5 (25%) Not daily but more than twice every week 15 (75%)	Once daily 12 (52.2%) Not daily but more than twice every week 11 (47.8%)	
Main techniques for faecal evacuation	Suppository 38 (88.4%) Digital stimulation 7 (16.3%) Manual evacuation 10 (23.3%) Spontaneous 2 (4.7%)	Suppository 20 (100%) Digital stimulation 6 (30%) Manual evacuation 8 (40%)	Suppository 18 (78.3%) Digital stimulation 1 (2.3%) Manual evacuation 2 (4.6%) Spontaneous 2 (4.6%)	
Supplementary interventions	Abdominal massage 25 (58.1%) Digital anus–rectal stimulation 21 (48.8%) Digital evacuation 4 (9.3%) Taking cathartic drug 4 (9.3%)	Abdominal massage 10 (50%) Digital anus–rectal stimulation 7 (35%) Digital evacuation 2 (10%) Taking cathartic drug 1 (5%)	Abdominal massage 15 (65.2%) Digital anus–rectal stimulation 14 (60.9%) Digital evacuation 2 (8.7%) Taking cathartic drug 3 (13%)	
Timing of bowel care	Morning 4 (9.3%) Afternoon 27 (62.8%) Evening 9 (20.9%) Inconsistent 3 (7%)	Morning 1 (5%) Afternoon 14 (70%) Evening 5 (25%)	Morning 3 (13%) Afternoon 13 (56.5%) Evening 4 (17.4%) Inconsistent 3 (13%)	
Location during evacuation	Bed 19 (44.2%) Toilet seat 8 (18.6%) Potty chair 16 (37.2%)	Bed 12 (60%) Toilet seat 2 (10%) Potty chair 6 (30%)	Bed 7 (30.4%) Toilet seat 6 (26.1%) Potty chair 10 (43.5%)	
Degree of assistance needed	Need all help 23 (53.5%) Need partial help 10 (23.3%) Independent completion 9 (20.9%) Need special help 1 (2.3%)	Need all help 19 (95%) Need special help 1 (5%)	Need all help 4 (17.4%) Need partial help 10 (43.5%) Independent completion 9 (39.1%)	
Abdominal discomfort	27 (62.8%)	12 (60%)	15 (65.2%)	
Constipation	29 (67.4%)	14 (70%)	15 (65.2%)	
Bloating symptom	32 (74.4%)	16 (80%)	16 (69.6%)	
Flatus incontinence	38 (88.4%)	18 (90%)	20 (87%)	
Lifestyle alteration due to NBD	Major impact 25 (58.1%) Some impact 15 (43.9%) Little impact 3 (7%)	Major impact 12 (60%) Some impact 6 (30%) Little impact 2 (10%)	Major impact 13 (56.5%) Some impact 9 (39.1%) Little impact 1 (4.3%)	
Top 3 complication desired to be solved	Neurogenic bowel dysfunction 42 (97.7%) Neurogenic bladder 36 (83.7%) Sexual dysfunction 19 (44.2%) Spasm 14 (32.6%) Neuralgia 8 (18.6%)	Neurogenic bowel dysfunction 19 (95%) Neurogenic bladder 16 (80%) Sexual dysfunction 8 (40%) Spasm 7 (35%) Neuralgia 3 (15%)	Neurogenic bowel dysfunction 23 (100%) Neurogenic bladder 20 (87%) Sexual dysfunction 11 (47.8%) Spasm 7 (30.4%) Neuralgia 5 (21.7%)	

More than half of the patients’ bowel care time occurred in the afternoon (62.8%), and the remaining patients’ bowel care time occurred in the evening (20.9%) and morning (9.4%). The location of bowel care was bed (44.2%), a potty chair (37.2%) or a toilet seat (18.8%). A total of 53.5% of patients needed full assistance during defecation time, 25.6% patients needed partial help, and 20.9% patients could defecate independently. Of the patients, 62.8% had abdominal discomfort, 67.4% had constipation, 74.4% had bloating, and 88.4% had flatus incontinence. The top 3 complications that patients wanted to resolve were NBD (100%), neurogenic bladder (83.7%), and sexual dysfunction (44.2%).

Patients with quadriplegia and SCI had a significantly higher BMI (23.586 ± 3.35) than did patients with paraplegia and SCI (22.697 ± 2.31) (p < 0.001). There were significant differences between the two groups in HDL, UREA and CRP (p < 0.001) (Table 3). Compared with patients with paraplegia and SCI, patients with quadriplegia and SCI had longer defecation times, higher NBD scores, lower defecation frequencies, and needed more supplementary interventions to complete bowel care.

For the majority of patients with quadriplegia and SCI, defecation occurred in bed. Almost all patients with quadriplegia and SCI required total help to complete bowel care, but most patients with paraplegia and SCI could finish bowel care independently or needed partial help only. Most patients with SCI reported abdominal discomfort, such as constipation, bloating, and flatus incontinence. More than half of the patients in both groups reported a serious impact of these complications on their lifestyle, and the most common complication that they wanted to resolve was NBD.

### Composition of the gut microbiome of healthy males and males with chronic traumatic complete SCI

To exclude the effect of gender on gut microbiota results, we selected 23 healthy male individuals and 43 male patients with SCI to perform a comparative analysis. Demographics and serum biomarkers between healthy males and patients with chronic traumatic complete SCI are shown in Table 2.

**Table 2:** Demographics and serum biomarkers between male healthy and patients with chronic traumatic complete SCI

	Health male	SCI-male	p
N	23	43	
AGE	40 ± 9.03	39.9 ± 10.57	0.998
BMI	24.8 ± 2.677	23.11 ± 2.876	0.022
ALT	26.791 ± 16.367	26.2 ± 19.303	0.903
AST	23.848 ± 17.097	21 ± 9.8	0.429
GLU	4.343 ± 0.528	5.266 ± 1.964	0.033
TG	1.436 ± 1.319	1.928 ± 1.207	0.137
TCHO	3.695 ± 0.794	4.217 ± 1.005	0.038
HDL	0.9152 ± 0.2091	0.917 ± 0.163	0.974
LDL	2.177 ± 0.596	2.617 ± 0.701	0.005
UREA	4.416 ± 1.224	4.403 ± 1.14	0.966
CR	64.3 ± 12.701	60.7 ± 11.8	0.265
UA	309 ± 69.81	378.1 ± 64.93	0.001

**Fig. 1:**
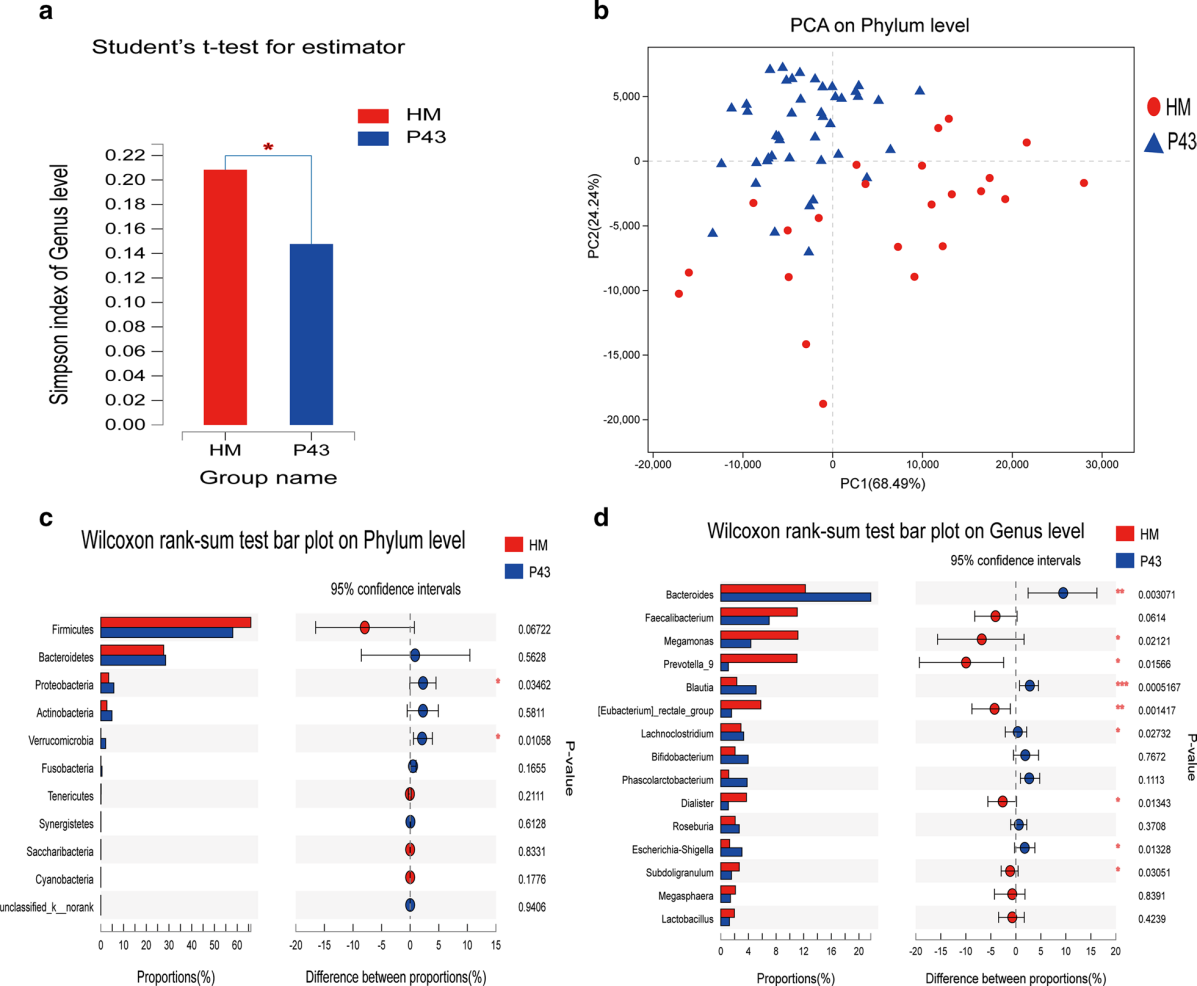
Diversity and taxonomic analysis in the healthy male and SCI groups. **a** At the genus level, the Simpson index showed a significant difference between the healthy male and SCI groups (p = 0.03635). **b** Plot of principal coordinate analysis (PCA) on the phylum level of the faecal microbiota based on the unweighted UniFrac metric in healthy male and SCI groups. STAMP analysis at the phylum and genus levels showed differences between the healthy male and SCI groups. Two of the top 15 phyla (**c**) and 9 of the top 15 genera (**d**) showed a significant difference (p < 0.05) between the two groups (Welch’s t-test)

Briefly, a total of 2,247,802 sequences were obtained. Reads were clustered in OTUs at 97% identity. Rarefaction curves showed clear asymptotes, and the Good’s coverage for the observed OTUs was 99.88%. Together, these findings indicated a near-complete sampling of the community. A total of 798 OTUs were recognised. No significant differences in OTU abundance (ace or chao1 index) were observed between healthy male and SCI populations. At the genus level, the Simpson alpha-diversity index showed a significant difference between the two groups (p = 0.03635) (Fig. 1a and Additional file 1). This finding indicated a decrease in intestinal flora diversity in patients with SCI.

The PCA on phylum level, the NIMDS on the out level, and the genus level of beta-diversity analysis revealed significant differences in bacterial community composition between the two groups. ANOSIM/Adonis revealed significant differences in the structure of the gut microbiota between the two groups (p < 0.05) (Additional file 2). PLS-DA revealed significant differences in bacterial community composition between the two groups on the phylum level (p < 0.05) (Fig. 1b).

STAMP analysis indicated that 9 of the top 15 genera showed a significant difference (p < 0.05) between the two groups (Welch’s *t* test). The abundance of Megamonas, Prevotella_9, [Eubacterium]_rectale_group, Dialister, and Subdoligranulum in the healthy male group was significantly higher than that in the SCI group (p < 0.05, Mann–Whitney U test). The abundance of Bacteroides, Blautia, Lachnoclostridium, and Escherichia-Shigella in the SCI group was significantly higher than that in the healthy male group (p < 0.05, Mann–Whitney U test) (Fig. 1c, d). LEfSe analysis (LDA threshold of 2) revealed that Veillonellaceae and Prevotellaceae were significantly enriched in the SCI group, whereas Bacteroidaceae and Bacteroides were significantly enriched in the healthy male group.

**Fig. 2:**
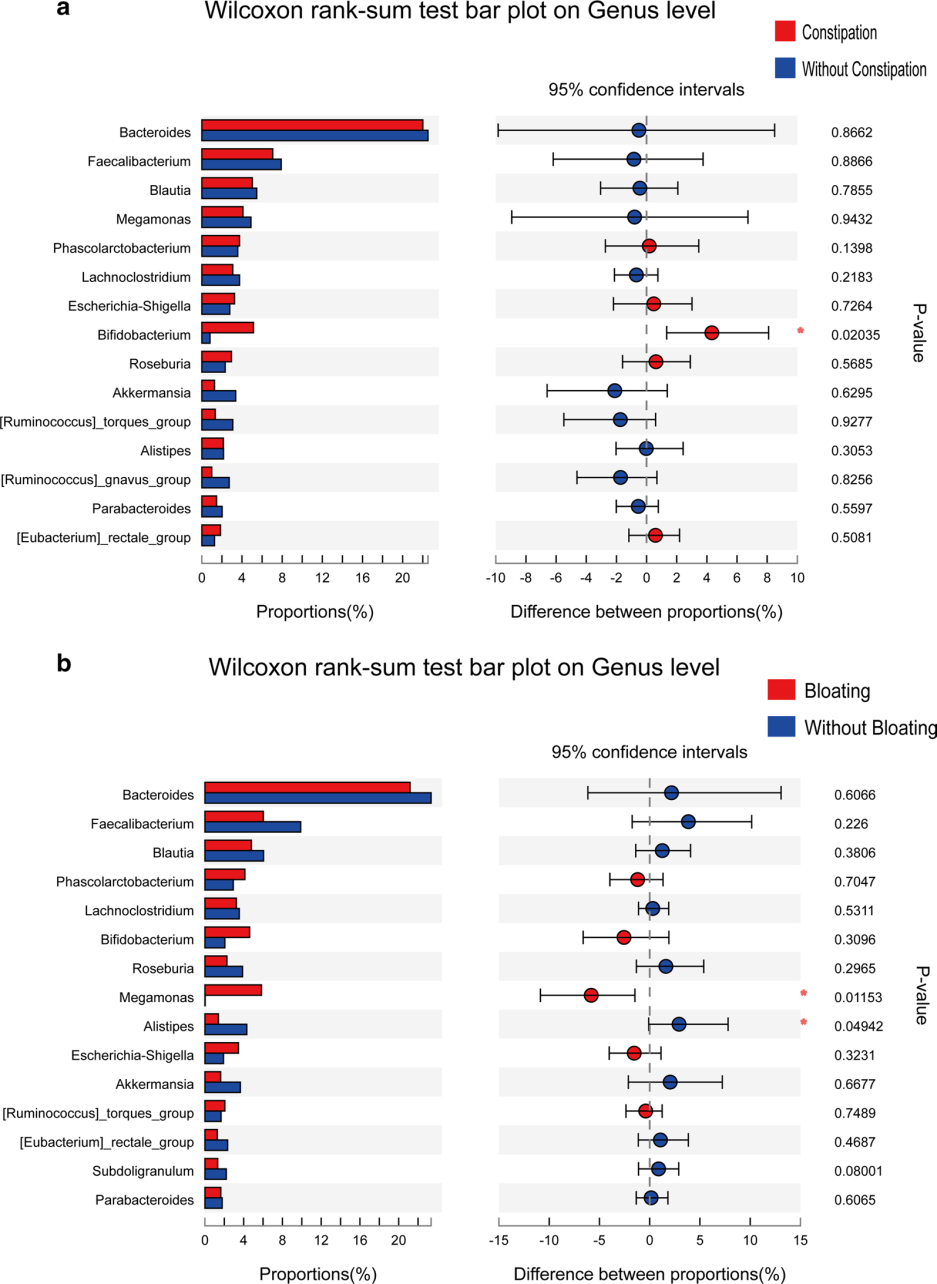
STAMP analysis of NBD symptoms. **a** STAMP analysis showed a significant difference (p < 0.05) between the two groups (Welch’s t-test) in Bifidobacterium at the genus level. **b** STAMP analysis showed that Megamonas was significantly higher (p < 0.05) in the bloating group and that Alistipes was significantly higher (p < 0.05) in the without bloating group at the genus level

According to NBD constipation symptoms, we divided patients with SCI into the constipation group and without constipation group. STAMP analysis showed a significant difference (p < 0.05) between the two groups (Welch’s t-test) in Bifidobacterium on the genus level (Fig. 2a). We also divided patients with SCI into the bloating group and without bloating group according to bloating symptoms. STAMP analysis showed that Megamonas was significantly higher (p < 0.05) in the bloating group, and Alistipes was significantly higher (p < 0.05) in the without bloating group on the genus level (Fig. 2b).

We selected the following environmental factors for RDA analysis: BMI, AGE, ALT, AST, GLU, TG, TCHO, HDL, LDL, UREA, CR, and UA. One-way ANOVA showed statistically significant differences in BMI, GLU, TCHO, LDL and UA between the two groups (p < 0.05) (Additional file 3). RDA/CCA showed that GLU (p = 0.017, r^2^ = 0.1315), HDL (p = 0.028, r^2^ = 0.1121), CR (p = 0.017, r^2^ = 0.1349) significantly affected bacterial composition at the phylum level. In the top 20 genera, BMI (p = 0.04, r^2^ = 0.0971), GLU (p = 0.044, r^2^ = 0.108) and HDL (p = 0.001, r^2^ = 0.3044) significantly affected bacterial composition (Additional files 4, 5). We found that serum biomarkers GLU, HDL and CR had significant correlations with microbial community structure (p < 0.05).

**Fig. 3:**
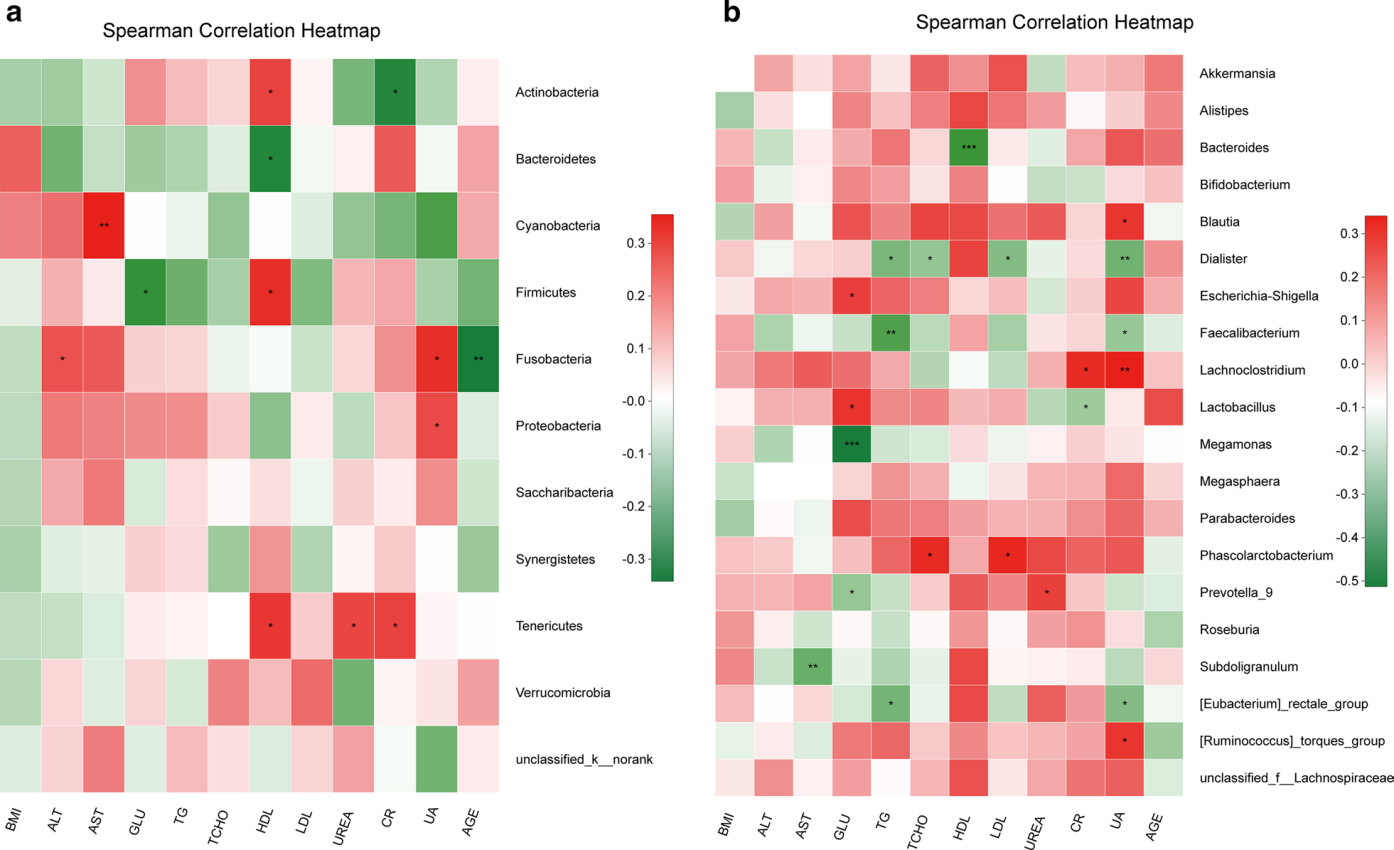
Correlation heatmap analysis of different environmental factors on the community composition of the healthy male and SCI groups at the phylum level (**a**) and genus level (**b**)

Correlation heatmap analysis of the relationship between different environmental factors and the community composition of the two groups showed that Proteobacteria was positively correlated with UA (Pearson r = 0.26, p = 0.035). Cyanobacteria was positively correlated with AST (Pearson r = 0.355, p = 0.003). Fusobacteria was negatively correlated with AGE (Pearson r = − 0.342, p = 0.005) and positively correlated with UA (Pearson r = 0.311, p = 0.011) and ALT (Pearson r = 0.244, p = 0.048). Bacteroidetes was negatively correlated with HDL (Pearson r = − 0.312, p = 0.011). Tenericutes was positively correlated with HDL (Pearson r = 0.292, p = 0.017), UREA (Pearson r = 0.266, p = 0.031) and CR (Pearson r = 0.275, p = 0.026). Actinobacteria was negatively correlated with CR (Pearson r = − 0.309, p = 0.012) and positively correlated with HDL (Pearson r = 0.273, p = 0.027). Finally, Firmicutes was positively correlated with HDL (Pearson r = 0.315, p = 0.010) and negatively correlated with GLU (Pearson r = − 0.279, p = 0.023) at the phylum level (Fig. 3a). In the top 20 genera, Bacteroides was negatively correlated with HDL (Pearson r = − 0.418, p < 0.001). Megamonas was negatively correlated with GLU (Pearson r = − 0.513, p < 0.001). Blautia was positively correlated with UA (Pearson r = 0.274, p = 0.026). Dialister was negatively correlated with UA, LDL, TG and TCHO (Pearson r = − 0.32, p = 0.009; r = − 0.289, p = 0.019; r = − 0.258, p = 0.037; and r = − 0.303, p = 0.013, respectively). Escherichia–Shigella was positively correlated with GLU (Pearson r = 0.245, p = 0.047). Faecalibacterium was negatively correlated with UA (Pearson r = − 0.252, p = 0.041) and TG (Pearson r = − 0.384, p = 0.001). Lachnoclostridium was positively correlated with UA (Pearson r = 0.341, p = 0.005) and CR (Pearson r = 0.297, p = 0.015). Lactobacillus was negatively correlated with CR (Pearson r = − 0.244, p = 0.048) and positively correlated with GLU (Pearson r = 0.278, p = 0.024). Phascolarctobacterium was positively correlated with LDL (Pearson r = 0.315, p = 0.010) and TCHO (Pearson r = 0.304, p = 0.013). Prevotella_9 was negatively correlated with GLU (Pearson r = − 0.257, p = 0.037) and positively correlated with UREA (Pearson r = 0.248, p = 0.045). Subdoligranulum was negatively correlated with AST (Pearson r = − 0.333, p = 0.006). [Eubacterium]_rectale_group was negatively correlated with UA (Pearson r = − 0.288, p = 0.019) and TG (Pearson r = − 0.314, p = 0.010). Finally, [Ruminococcus]_torques_group was positively correlated with UA (Pearson r = 0.274, p = 0.026) (Fig. 3b and Additional files 6, 7).

### Comparison of the gut microbiome in the quadriplegia and paraplegia groups

We divided the 43 patients with SCI into a quadriplegia group (n = 20) and paraplegia group (n = 23); the characteristics and neurogenic bowel management of these groups are shown in Tables 1 and 3. The defecation time of patients with quadriplegia (41.789 ± 19.29 min) was significantly longer than that of patients with paraplegia (30 ± 13.94 min) (p = 0.026).

**Table 3:** Demographics and serum biomarkers male patients with chronic traumatic complete SCI

	SCI-male	Sci-cervical	Sci-thoracolumbar	p
N	43	20	23	
AGE	39.9 ± 10.57	41.5 ± 8.30	38.5 ± 12.04	0.369
BMI	23.11 ± 2.876	23.586 ± 3.35	22.697 ± 2.31	< 0.001
ALT	26.2 ± 19.303	23.09 ± 11.04	28.16 ± 24.132	0.487
AST	21 ± 9.8	21 ± 9.32	22 ± 10.2	0.796
GLU	5.266 ± 1.964	5.766 ± 2.68	4.83 ± 0.747	0.125
TG	1.928 ± 1.207	2.0325 ± 1.259	1.837 ± 1.15	0.607
TCHO	4.217 ± 1.005	4.072 ± 1.067	4.34 ± 0.93	0.39
HDL	0.917 ± 0.163	0.846 ± 0.137	0.979 ± 0.159	0.007
LDL	2.617 ± 0.701	2.68 ± 0.658	2.69 ± 0.74	0.965
UREA	4.403 ± 1.14	3.956 ± 0.975	4.791 ± 1.13	0.016
CR	60.7 ± 11.8	61.2 ± 11	60.3 ± 12.3	0.815
UA	378.1 ± 64.93	380.75 ± 60.99	375.7 ± 68.08	0.806
CRP	7.78 ± 10.31	11.2 ± 13.45	4.79 ± 4.674	0.042

**Fig. 4:**
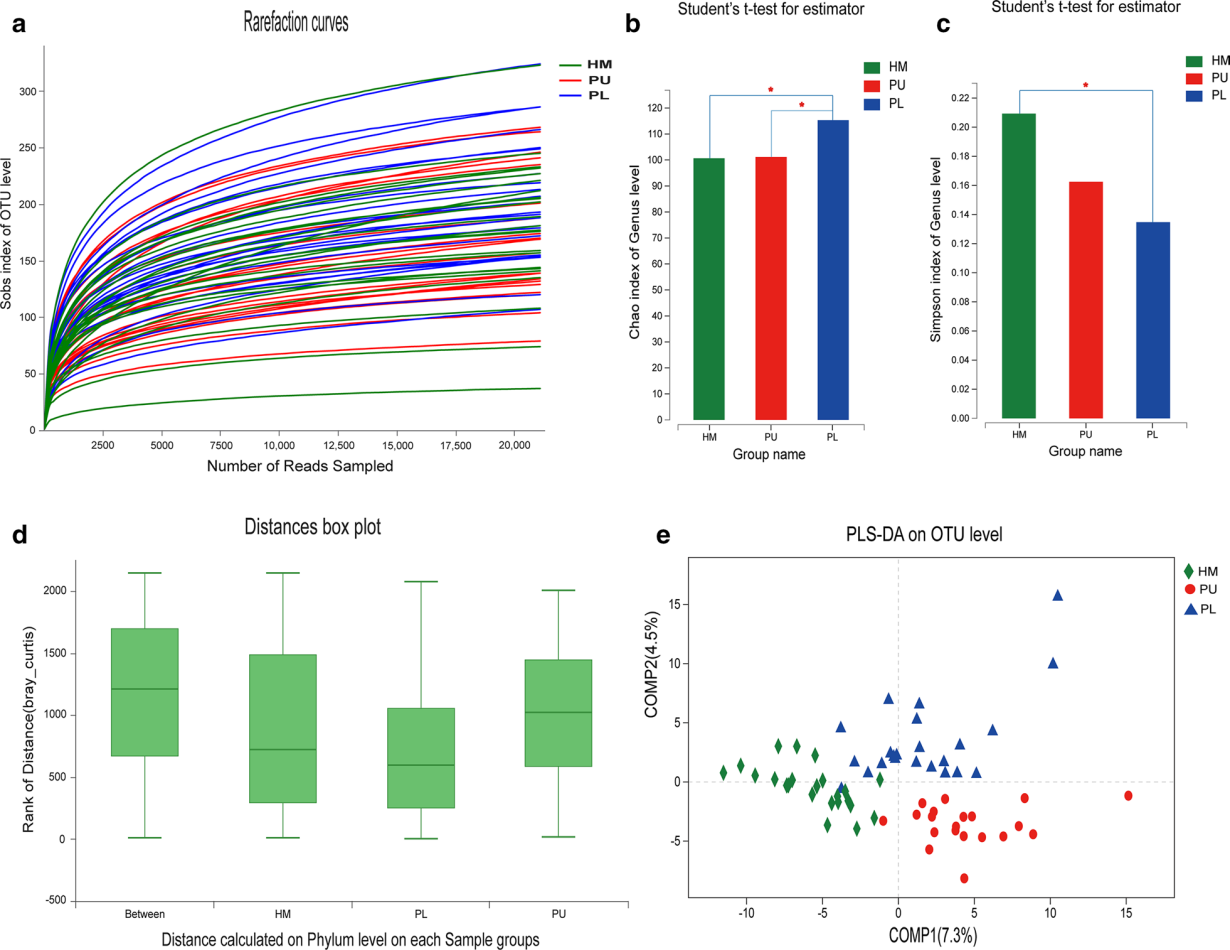
Diversity and taxonomic analysis in the healthy male, quadriplegia (PU) and paraplegia (PL) SCI groups. **a** Sobs index of rarefaction curves for the healthy male, quadriplegia and paraplegia groups of samples based on OTUs detected using a similarity threshold of 97%. **b** Significant differences in the genus chao index (**b**) and Simpson index (**c**) were observed between the three populations (p < 0.05). **d** ANOSIM/Adonis of beta-diversity analysis revealed significant differences in the structure of the gut microbiota among the three groups (p = 0.001, r^2^ = 0.233) at the phylum level. **e** PLS-DA revealed that there were significant differences in bacterial community composition between the three groups at the OTU, phylum and genus levels (PU and PL represent patients with quadriplegia and paraplegia, respectively)

Rarefaction curves showed clear asymptotes, and the Good’s coverage for the observed OTUs was 99.88%; together, these findings indicated a near-complete sampling of the community (Fig. 4a). No significant difference in OTU abundance (ace or chao index) was observed between the three groups. Based on the chao index, a significant difference in the genus abundance was observed between the quadriplegia group and paraplegia group (p = 0.02922) and the healthy male group and paraplegia group (p = 0.02919). These findings indicated a difference in community richness between the two groups (Fig. 4b). The Simpson index of the healthy male group was significantly higher than that of the paraplegia group (p = 0.04094) at the genus level, indicating a decrease in intestinal flora diversity in patients with paraplegia and SCI (Fig. 4c and Additional files 8, 9, 10).

ANOSIM/Adonis of beta-diversity analysis revealed significant differences in the structure of the gut microbiota between the three groups (p = 0.001, r^2^ = 0.233) at the phylum level (Fig. 4d and Additional file 11). PLS-DA revealed significant differences in bacterial community composition between the three groups on the OTU, phylum and genus levels (Fig. 4e).

**Fig. 5:**
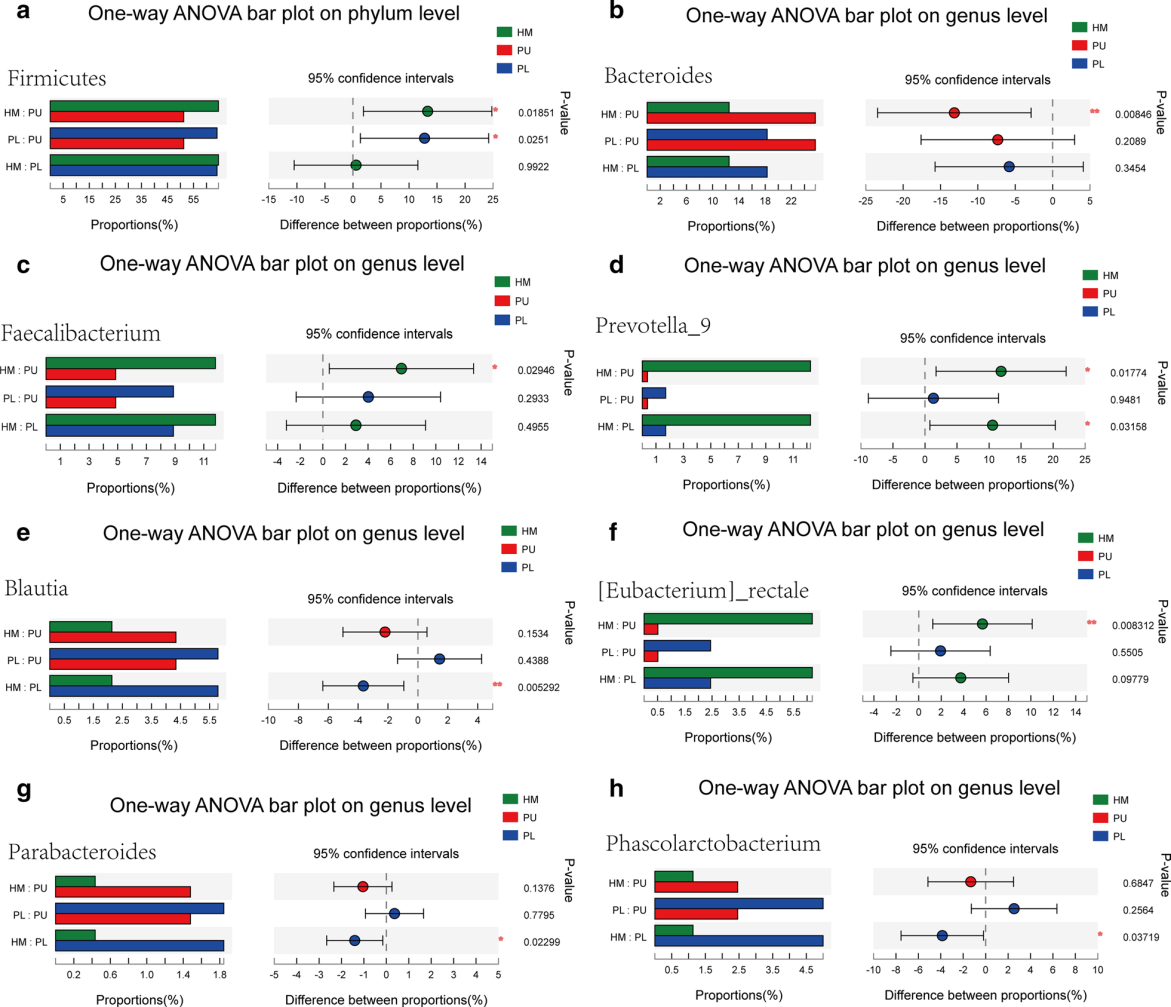
STAMP analysis indicated significant differences at the phylum (**a**) and genus (**b**–**h**) levels between the three groups

STAMP analysis identified 8 OTUs showing a significant difference (p < 0.05) between the three groups (Welch’s t-test) in the top 15 OTUs. Eight of the top 15 genera showed a significant difference (p < 0.05) between the three groups (Welch’s t-test). The abundance of Firmicutes in the paraplegia group and healthy male group was significantly higher than that in the quadriplegia group (p = 0.0251; p = 0.0185). In the top 15 genera, the abundance of Bacteroides, Faecalibacterium, Blautia, Prevotella_9, Phascolarctobacterium, Parabacteroides, and [Eubacterium]_rectale showed a significant difference between the three groups (p < 0.05, one-way ANOVA) (Fig. 5).

The 16 environmental factors selected for RDA analysis in the quadriplegia and paraplegia groups were as follows: BMI, ALT, AST, GLU, TG, TCHO, HDL, LDL, UREA, CR, UA, AGE, COURSE, CRP, NBD score, and defecation time. One-way ANOVA showed statistically significant differences in BMI, HDL, UREA, APOA1 and defecation time between the two groups (Table 3, Additional file 12).

RDA/CCA showed that GLU (p = 0.014, r^2^ = 0.1969), HDL (p = 0.009, r^2^ = 0.2274), and CR (p = 0.006, r^2^ = 0.2306) significantly affected bacterial composition at the phylum level. At the OTU level, TG (p = 0.042, r^2^ = 0.2192), CR (p = 0.007, r^2^ = 0.2388), and defecation time (p = 0.022, r^2^ = 0.2009) significantly affected bacterial composition. At the genus level, HDL (p = 0.001, r^2^ = 0.4675) and CR (p = 0.001, r^2^ = 0.3209) significantly affected bacterial composition (Additional files 13, 14).

Correlation heatmap analysis of the relationship between different environmental factors and the community composition of quadriplegia and paraplegia groups showed that Alistipes was negatively correlated with defecation time (Pearson r = − 0.363, p = 0.017) and negatively correlated with COURSE (Pearson r = − 0.375, p = 0.013). Bacteroides was negatively correlated with HDL (Pearson r = − 0.684, p < 0.001) and positively correlated with TG (Pearson r = 0.354, p = 0.020) and CR (Pearson r = 0.312, p = 0.042).

Lachnoclostridium was negatively correlated with HDL (Pearson r = − 0.329, p = 0.031) and positively correlated with AST (Pearson r = 0.305, p = 0.047), UA (Pearson r = 0.358, p = 0.018) and CR (Pearson r = 0.403, p = 0.007). Lactobacillus was positively correlated with TCHO (Pearson r = 0.324, p = 0.034), AGE (Pearson r = 0.357, p = 0.019) and CRP (Pearson r = 0.431, p = 0.007). Megamonas was positively correlated with the NBD score (Pearson r = 0.309, p = 0.044). Megasphaera was positively correlated with defecation time (Pearson r = 0.373, p = 0.014) and COURSE (Pearson r = 0.327, p = 0.032).

Prevotella_9 was positively correlated with the NBD score (Pearson r = 0.315, p = 0.040), LDL (Pearson r = 0.302, p = 0.049) and HDL (Pearson r = 0.32, p = 0.036). Roseburia was negatively correlated with CRP (Pearson r = − 0.341, p = 0.025). [Eubacterium]_rectale_group was negatively correlated with CRP (Pearson r = − 0.337, p = 0.027) and positively correlated with UREA (Pearson r = 0.361, p = 0.017).

**Fig. 6:**
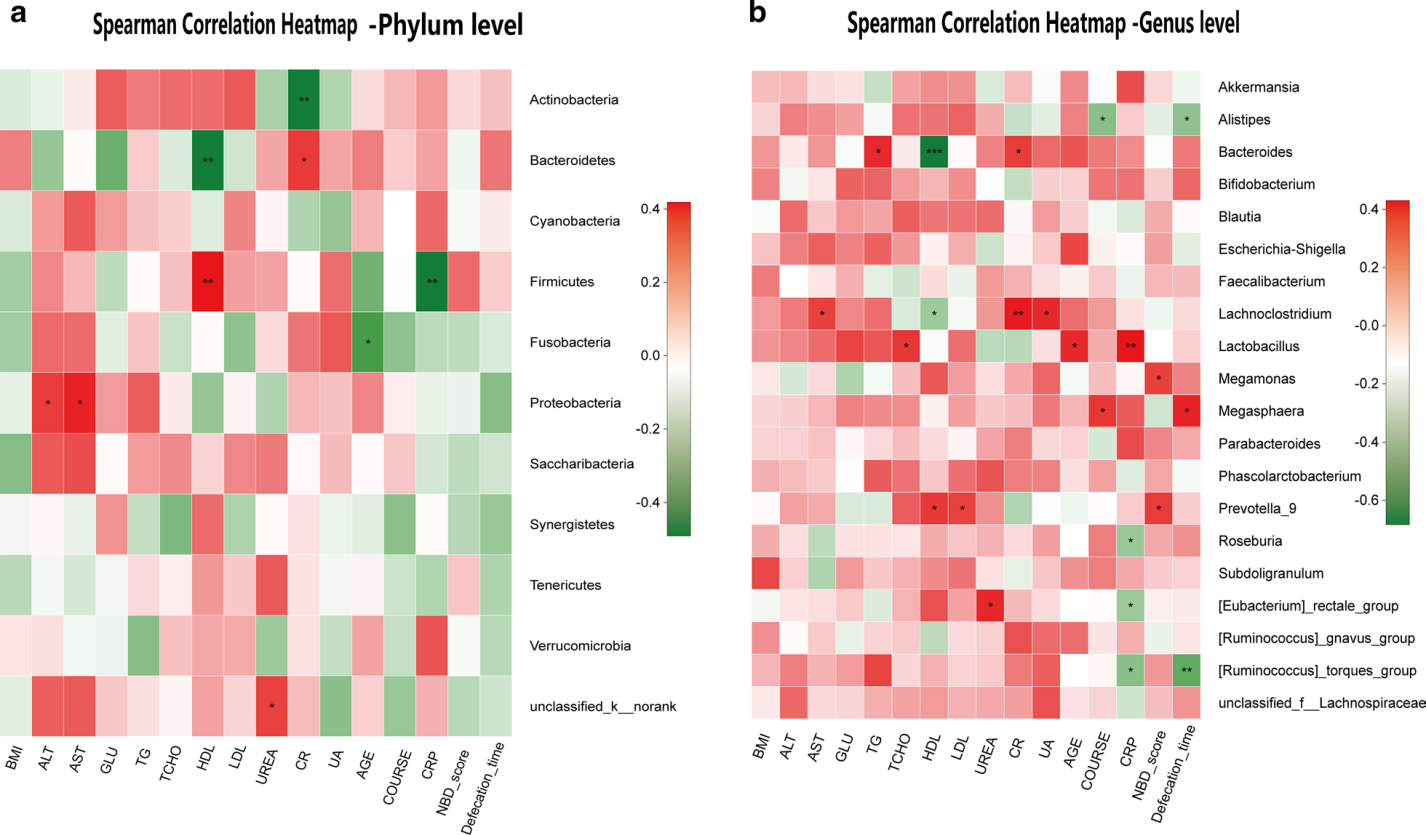
Correlation heatmap analysis of different environmental factors on the community composition of the quadriplegia and paraplegia groups at the phylum level (**a**) and genus level (**b**)

[Ruminococcus]_torques_group was negatively correlated with CRP (Pearson r = − 0.381, p = 0.012) and defecation time (Pearson r = − 0.47, p = 0.001). At the phylum level, Firmicutes was negatively correlated with CRP (Pearson r = − 0.491, p = 0.001) and positively correlated with HDL (Pearson r = 0.419, p = 0.005). Actinobacteria was negatively correlated with CR (Pearson r = − 0.486, p = 0.001). Bacteroidetes was negatively correlated with HDL (Pearson r = − 0.486, p = 0.001) and positively correlated with CR (Pearson r = 0.327, p = 0.032). Fusobacteria was negatively correlated with AGE (Pearson r = − 0.365, p = 0.016). Proteobacteria was positively correlated with ALT (Pearson r = 0.325, p = 0.034) and AST (Pearson r = 0.375, p = 0.013). Based on our results, at the genus and OTU levels, HDL, LDL, CR, UA, and AGE had an effect on the intestinal microbiota of the two groups (Fig. 6, Additional files 15, 16).

## Discussion

In this study, we compared the gut microbiome between healthy adult males and male patients with chronic traumatic complete SCI. The neurogenic bowel management of patients with SCI in our centre was first reported through cross-sectional interviews. Then, we explored the association between the gut microbiota and environmental factors in the quadriplegia and paraplegia groups and analysed the correlation between the gut microbiota and neurogenic bowel symptoms. The neurogenic bowel symptoms in patients with SCI were related to some gut microbiota, which may help to explain the potential link between gut dysbiosis and NBD symptoms in patients with SCI.

Acute traumatic SCI (ASCI) used to appear mostly in adults (21–69 years), and the causes of injury were fall from height (37.5%) and traffic accidents (26.9%) [36, 37]. This finding was also consistent with the main causes of injury in this study, traffic accidents (37.2%) and fall from height (20.9%). The average age of patients with SCI in this study was 39.9 years old; thus, these patients were in the prime of their lives and were more susceptible to accidental injuries such as high-energy trauma. After a chronic course, the most common complication that patients hoped to resolve was NBD. The gut microbiota may be a potential target to help improve this problem.

Julia et al. illustrated the practice and outcomes of bowel care in a community of individuals with SCI in Malaysia [38]. Yasmeen reported that 43 of 50 adult patients with SCI in Pakistan had a history of occasional or regular faecal incontinence [39]. The prevalence of NBD in patients with SCI was 80% in a previous study, and 97.3% of patients with motor complete SCI had chronic NBD complaints in their study [10], which was similar to the number of patients who reported constipation in our study. Moreover, the patients in our study had to spend a substantial amount of time on defecation during their long course of SCI to address NBD.

Injury level has not been related to gastrointestinal complaints in patients with SCI, and no significant relationship was detected between the prevalence of gastrointestinal symptoms and the level of SCI in previous studies [40–42]. Most patients with quadriplegia have no active exercise capacity, but patients with paraplegia can complete all upper limb movements. The autonomic nervous system that supports the gastrointestinal tract in patients with quadriplegia and SCI remains relatively intact and has less of an impact on the function of the gastrointestinal tract. In patients with paraplegia, damage to the sympathetic centre or defecation centre would have a greater impact on intestinal function, which may explain the difference between the two groups.

Surveys among the SCI population often rank colorectal, bladder and sexual dysfunction as significant obstacles and prioritise the recovery of bowel function above the ability to walk [43–45]. In this study, the top 3 complications that patients wanted to resolve were NBD, neurogenic bladder, sexual dysfunction, and the gut microbiota, which may represent a potential solution.

Rajilić-Stojanović observed an increase in the phylum Bacteroidetes, which was reflected by increased Bacteroides at the genus level. The phylum Bacteroidetes encompasses a diverse, abundant group of Gram-negative commensal bacteria in the gut [46]. In our study, Bacteroides was significantly higher in the SCI group and negatively correlated with HDL; it was especially enriched in the quadriplegia group. HDL levels are positively correlated with the amount of exercise, and a lack of exercise can result in lower HDL levels [47]. Reduced exercise in patients with quadriplegia lowered HDL levels, and Bacteroides was increased in patients with quadriplegia. These findings indicated that Bacteroides may be a harmful flora and was associated with exercise and serum HDL levels.

In our study, Dialister was significantly higher in healthy males, and these bacteria were negatively correlated with UA, LDL, TG and TCHO. These elevated serum markers represent high blood lipids, which are harmful to health. Based on the present study, decreased Dialister in patients with SCI may aggravate the symptoms of NBD.

Ming et al. reported that the positive association between bean consumption and the Megamonas genus discovered in their study may implicate Megamonas as a beneficial microbe [48]. The abundance of Megamonas was decreased in the SCI group, negatively correlated with GLU and positively correlated with NBD scores, implying that Megamonas may have a positive effect on the body in terms of carbohydrate metabolism. A decreased abundance of Megamonas exacerbates NBD symptoms. Here, the relative abundance of Megamonas was significantly higher in the bloating group than in the non-bloating group, possibly because some carbohydrates in food cannot be digested and absorbed by intestinal digestive enzymes. However, these carbohydrates can be metabolised by Megamonas in the large intestine, producing gas and causing bloating, exacerbating NBD symptoms.

Alistipes was reportedly altered in irritable bowel syndrome with an animal-based diet or vegetable consumption [49–51]. Alistipes was also associated with the phenotype of frequently recurrent abdominal pain [49]. In the present study, the relative abundance of Alistipes was significantly decreased in the bloating group, and Alistipes was negatively correlated with defecation time. Thus, Alistipes may be a factor that affects defecation time between the quadriplegia and paraplegic groups because the abundance of Alistipes and defecation time in the quadriplegia group were significantly higher than those in the paraplegia group. However, a comprehensive understanding of the biochemical and functional roles of Alistipes is lacking.

Members of the Bifidobacterium genus are important bacterial inhabitants of the human gut across the lifespan, and their beneficial health effects have been well documented [52, 53]. However, certain species of Bifidobacterium are associated with decreased intestinal permeability. In our study, the abundance of Bifidobacterium and Bacteroides was increased in the SCI group, possibly because the increased bacterial translocation effect of Bacteroides played a more important role than that of Bifidobacterium. The relative abundance of Bifidobacterium was decreased in SCI group, supporting the role of Bifidobacterium as a beneficial microbe. The relative abundance of Bifidobacterium was significantly higher in constipation SCI group than without constipation SCI group in our study, it may because the Bifidobacterium can liberate short-chain fatty acids and gases by collaborating with other species over the chronic course of SCI [54, 55]. Another possible explain is Bifidobacterium promotes bloating symptoms in the two-way regulation of diarrhoea and constipation symptoms [52, 53]. More investigation is needed to determine the interaction of this bacteria with NBD symptoms.

We also examined the association between serum biomarkers and the gut microbiota. Prevotella is considered a beneficial microbe, but it is also linked with chronic inflammatory conditions [56–59]. In our study, the Prevotella level was decreased in the SCI group and was negatively correlated with GLU, which implies that Prevotella has a positive effect on the body in terms of carbohydrate metabolism, supporting the role of Prevotella as a beneficial microbe.

Li et al. reported that the male/female ratio of ASCI is 3.1/1 [36]. Most patients with chronic traumatic complete SCI admitted to our centre were males; thus, we chose male patients as our research subjects. A strong point of this study was the inclusion of only male patients with complete SCI. This approach excluded the probable confounding effects of gender and residual nerves on gut functions; therefore, other incomplete injuries were not included in this study. Individual diet-associated flora differences could not be determined and remain a major weakness of this study. Gut microbes produce neuroactive metabolites (short-chain fatty acids and choline) and neurotransmitters (γ-aminobutyric acid, serotonin, dopamine, and acetylcholine), which can affect autonomic nervous system function by activating vagal afferent nerve fibres and various modes of communication in the intestines [3, 5]. SCI disrupts the autonomic nervous system, impairing its ability to coordinate organ function throughout the body [9, 12, 27, 29]. In our study, gut dysbiosis, NBD and some serum biomarkers were altered in patients with SCI, and gut dysbiosis was correlated with serum biomarkers and NBD symptoms. These issues occurred simultaneously after injury and affected each other; inactivity in patients with SCI may be another factor affecting these issues [47]. Continuing analyses of genomic and metagenomic changes in the gut microbiota will allow scientists to map the dynamic patterns of dysbiosis caused by SCI and clarify how serum biomarkers affect the SCI pathological mechanism or NBD through the gut microbiota. Unfortunately, only male patients with SCI were enrolled in our study. Therefore, future studies should include female patients to identify gender disparities. Further work, including animal experiments and longitudinal human studies, will be needed to determine the cause–effect relationship between the gut microbiota and SCI. Determining the role of the gut microbiota in the progression or maintenance of SCI may lead to novel interventional approaches that alter or restore healthy gut bacterial composition or the identification of microbial metabolites that are protective against SCI.

## Conclusions

In conclusion, this study presents a comprehensive landscape of the gut microbiota in adult male patients with traumatic complete SCI and documents their neurogenic bowel management. We found a difference in faecal flora between healthy adult males and male patients with SCI. Moreover, the dysbiosis of patients with SCI was correlated with serum biomarkers and NBD symptoms.

## Additional files

**Additional file 1.** Alpha-diversity index inter-group difference test chart between healthy male and SCI cohorts.

**Additional file 2.** ANOSIM/Adonis on OUT, phylum and genes level revealed significant differences in the structure of gut microbiota among the healthy male and SCI groups (p < 0.05).

**Additional file 3.** VIF of selected environmental factors by the functions of envfit (permu = 999) and vif.cca among the healthy male and SCI groups.

**Additional file 4.** RDA/CCA showed that GLU, HDL, CR significantly affected bacterial composition in phylum level between the healthy male and SCI groups.

**Additional file 5.** RDA/CCA showed that BMI, GLU and HDL significantly affected bacterial composition in genus level between the healthy male and SCI groups.

**Additional file 6.** Correlation heatmap analysis chart of different environmental factors on the community composition of healthy male and SCI groups in phylum level.

**Additional file 7.** Correlation heatmap analysis chart of different environmental factors on the community composition of healthy male and SCI groups in genus level.

**Additional file 8.** Alpha-diversity index inter-group difference test chart between healthy male, quadriplegia and paraplegic SCI cohorts in OTUs level.

**Additional file 9.** Alpha-diversity index inter-group difference test chart between quadriplegia and paraplegic SCI cohorts in genus level.

**Additional file 10.** Alpha-diversity index inter-group difference test chart between healthy male and paraplegic SCI cohorts.

**Additional file 11.** ANOSIM/Adonis distances box plot on phylum level revealed significant differences in the structure of gut microbiota among the healthy male, quadriplegia and paraplegic SCI groups (p < 0.05).

**Additional file 12.** VIF of selected environmental factors by the functions of envfit (permu = 999) and vif.cca among the quadriplegia and paraplegic SCI groups.

**Additional file 13.** RDA/CCA showed that GLU, HDL, CR significantly affected bacterial composition in phylum level between quadriplegia and paraplegic SCI groups.

**Additional file 14.** RDA/CCA showed that HD, CR significantly affected bacterial composition in genus level between quadriplegia and paraplegic SCI groups.

**Additional file 15.** Correlation heatmap analysis chart of different environmental factors on the community composition of quadriplegia and paraplegic SCI groups in phylum level.

**Additional file 16.** Correlation heatmap analysis chart of different environmental factors on the community composition of quadriplegia and paraplegic SCI groups in genus level.

## Abbreviations

SCI: spinal cord injury
NBD: neurogenic bowel dysfunction
GLU: glucose
HDL: high density lipoprotein
LDL: low density lipoprotein
UA: uric acid
CR: creatinine
CPR: C-reactive protein
OTUs: operational taxonomic units
PLS-DA: partial least squares discrimination analysis
BMI: body mass index
APOA1: apolipoprotein A1
APOB: apolipoprotein B
ALT: alanine transaminase
AST: aspartate transaminase
TG: triglyceride
TCHO: total cholesterol
LPA: lipoprotein A
NEFA: non-esterified fatty acid
HCY: homocysteine
ASCI: acute spinal cord injury
LPS: lipopolysaccharide
AD: Alzheimer’s disease
HM: healthy male
PU: patient with quadriplegia and SCI
PL: patient with paraplegia and SCI
