# Supplementary figures and images for: Gut microbiota dysbiosis in male patients with chronic traumatic complete spinal cord injury
Source: J Transl Med. 2018 Dec 13;16:353. doi: 10.1186/s12967-018-1735-9 (PMC6293533; doi:10.1186/s12967-018-1735-9)

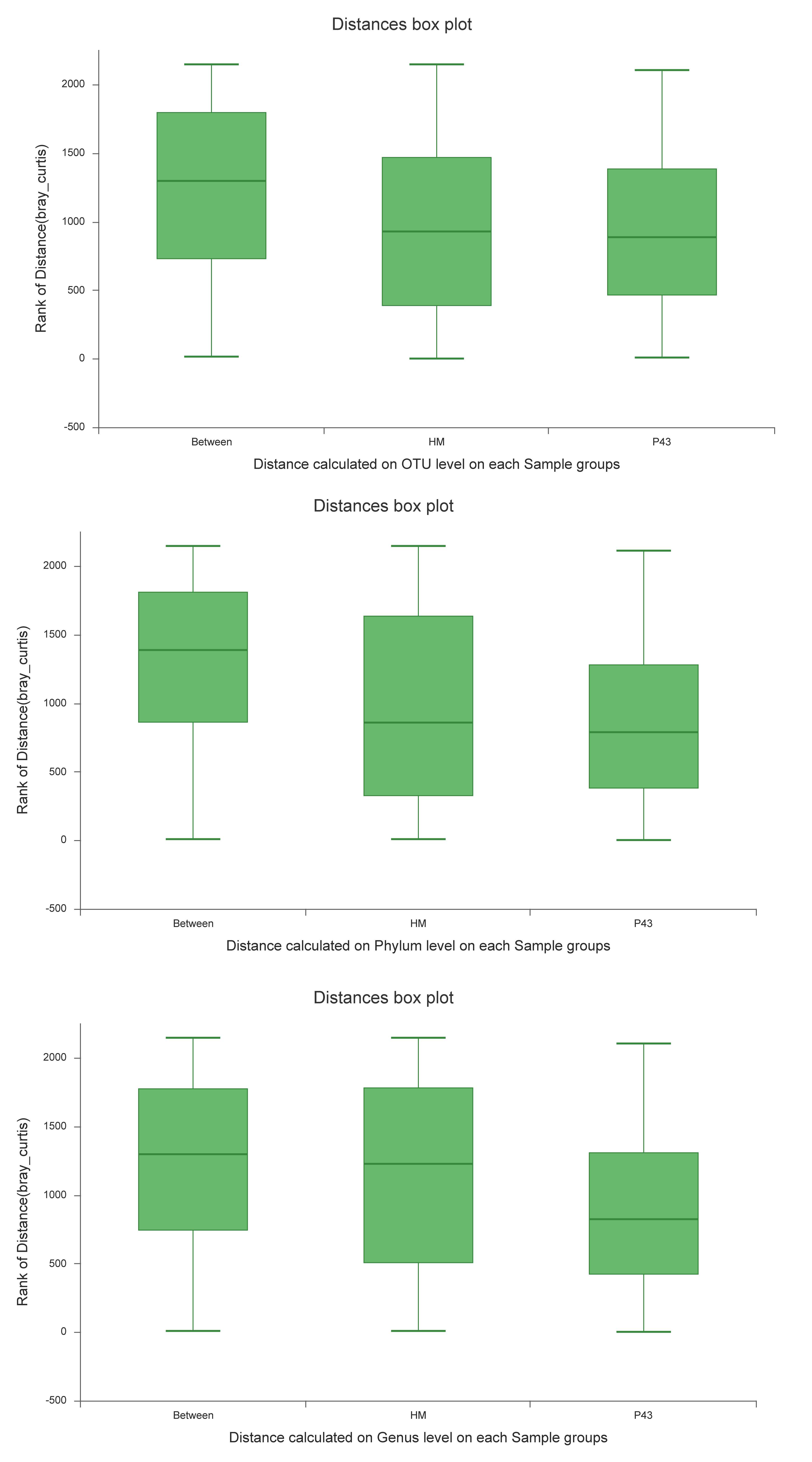

Supplement: Supplementary file 2 — Additional file 2. ANOSIM/Adonis on OUT, phylum and genes level revealed significant differences in the structure of gut microbiota among the healthy male and SCI groups (p < 0.05). [file 12967_2018_1735_MOESM2_ESM.tif]

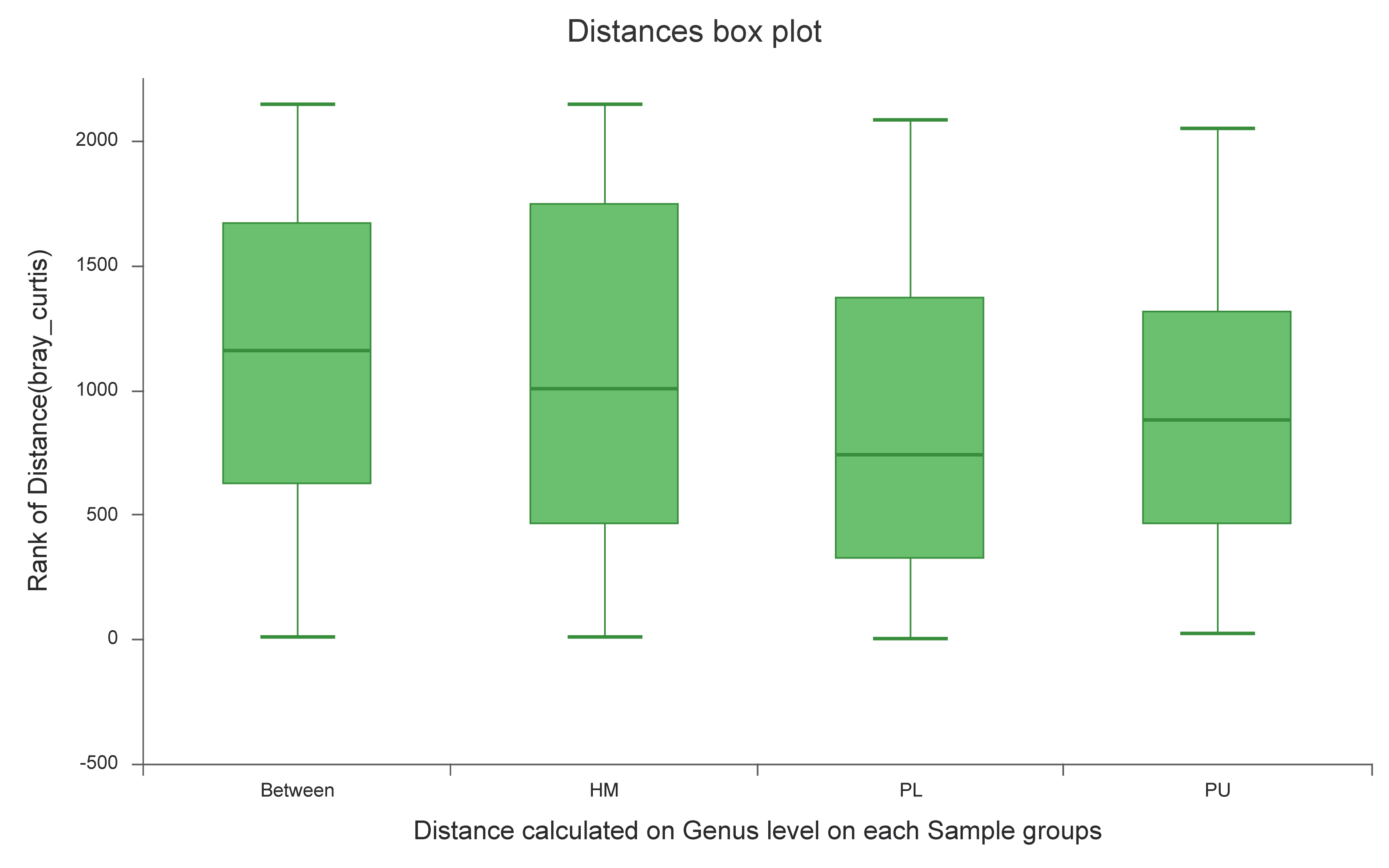

Supplement: Supplementary file 11 — Additional file 11. ANOSIM/Adonis distances box plot on phylum level revealed significant differences in the structure of gut microbiota among the healthy male, quadriplegia and paraplegic SCI groups (p < 0.05). [file 12967_2018_1735_MOESM11_ESM.tif]
